# Enhancing UV-B Protection and Abiotic Stress Tolerance in Tomato Plants: The Role of Silicon Nanoparticles in Photosynthetic Parameters, Pigments, and Secondary Metabolite Production

**DOI:** 10.3390/plants14162599

**Published:** 2025-08-21

**Authors:** Florina Copaciu, Cosmin-Alin Faur, Andrea Bunea, Loredana Leopold, Rodica Maria Sima, Mihai Andrei Lăcătuș, Andreea Lupitu, Cristian Moisa, Dana Maria Copolovici, Lucian Copolovici

**Affiliations:** 1 Faculty of Animal Science and Biotechnologies, University of Agricultural Sciences and Veterinary Medicine of Cluj-Napoca, 3-5 Manastur St., 400372 Cluj-Napoca, Romania; florina.copaciu@usamvcluj.ro (F.C.); cosmin-alin.faur@student.usamvcluj.ro (C.-A.F.); andrea.bunea@usamvcluj.ro (A.B.); mihai.lacatus@usamvcluj.ro (M.A.L.); 2 Faculty of Food Science and Technology, University of Agricultural Sciences and Veterinary Medicine of Cluj-Napoca, 3-5 Manastur St., 400372 Cluj-Napoca, Romania; dana.leopold@usamvcluj.ro; 3 Faculty of Horticulture and Business in Rural Development, University of Agricultural Sciences and Veterinary Medicine of Cluj-Napoca, 3-5 Manastur St., 400372 Cluj-Napoca, Romania; rodica.sima@usamvcluj.ro; 4 Institute for Interdisciplinary Research, Faculty of Food Engineering, Tourism and Environmental Protection, “Aurel Vlaicu” University of Arad, 2 Elena Dragoi St., 310330 Arad, Romania; pag.andreea@yahoo.com (A.L.); moisa.cristian@yahoo.com (C.M.); dana.copolovici@uav.ro (D.M.C.)

**Keywords:** *Solanum lycopersicum*, silicon nanoparticles, UV-B radiation, photosynthetic pigments, biogenic volatile organic compounds

## Abstract

Tomato fruit (*Solanum lycopersicum*) is a valuable agricultural crop worldwide due to its nutritional value and culinary applications, making it one of the most widely consumed vegetables in the human diet. However, excessive solar UV-B radiation represents a significant factor in decreasing productivity, marketable yields, and fruit quality in tomato crops by causing damage to both DNA and the photosynthetic system, as well as chlorophyll degradation. The application of silicon nanoparticles has been shown to increase tolerance to abiotic stressors, including enhanced UV-B radiation. Therefore, this study aims to evaluate the protective effects of foliar silicon nanoparticle (SiNP) application on photosynthetic parameters, photosynthetic pigments, and secondary metabolites under enhanced UV-B stress in tomato plants. Photosynthetic parameters (stomatal conductance to water vapor, net CO_2_ assimilation rate, transpiration rate, and intercellular CO_2_ molar fraction), biogenic volatile organic compounds (BVOCs), chlorophylls, and carotenoids were evaluated. The application of SiNPs showed beneficial effects on plants grown under ambient UV-B conditions, increasing photosynthetic parameters while also enhancing chlorophyll and carotenoid levels. In plants exposed to enhanced UV-B radiation, SiNP treatment helped to maintain and even improve photosynthetic parameters and stomatal function in leaves while also promoting the accumulation of photosynthetic pigments. Additionally, the application of SiNPs also resulted in a slightly higher content of lycopene and total carotenoids in tomato fruits.

## 1. Introduction

Environmental stresses remain the foremost constraint on agricultural productivity worldwide. Meta-analyses show that abiotic factors—most notably drought, salinity, temperature extremes, flooding, and high-energy radiation—can depress attainable yields by roughly 50–70%. Biotic challenges such as pathogens, insect pests, and weeds inflict additional losses, removing 20–40% of global production [1].

Plants are photoautotrophic organisms and, thus, must constantly adapt to changing environmental conditions [2]. Photosynthetic organisms harness solar radiation to convert light energy into chemical energy, a process that operates most efficiently within the photosynthetically active radiation (PAR) window of 400–700 nm [3]. Ultraviolet (UV) radiation, which represents roughly 1–8% of incident sunlight, is conventionally subdivided into three spectral bands: UV-A (315–400 nm), UV-B (280–315 nm), and UV-C (100–280 nm) [4]. UV-C is highly harmful, but fortunately, it is completely absorbed by the stratospheric ozone layer and does not reach the Earth’s surface. Of the ultraviolet radiation that reaches the Earth’s surface, 95% is UV-A, while the remaining 5% is UV-B. These two types of radiation affect the cells of all living organisms in different ways [5]. Among these, UV-A is the least harmful to organisms due to its lower energy and its ability to penetrate the ozone layer. However, the depletion in the stratospheric ozone layer, which is the primary attenuator of solar UV-B radiation, poses a threat to all living organisms [3,6]. Anthropogenic emissions of chlorine- and bromine-containing compounds exert the strongest influence on the ozone layer, with additional contributions from natural factors such as volcanic eruptions and climate-related variability [3,6,7].

Despite UV-B light comprising only a small fraction of the sun’s spectrum at the Earth’s surface, its elevated photon energy elicits significant reactions at the plant level. UV-B can cause direct photochemical damage to essential biomolecules (lipids, proteins, and nucleic acids) [6,7], induce the generation of reactive oxygen species, and disrupt the photosynthetic apparatus [8]. The major consequences lead to decreased photosynthetic efficiency, changes in plant morphology and physiology, modified secondary metabolite profiles, and, with prolonged or severe exposure, potential plant death [2,9,10]. Although numerous studies have been conducted on the effects of UV-B radiation and different mitigation procedures on plants [11,12], the application of silicon and its nanoparticles, along with their interaction with UV-B, remains an area of ongoing research.

Silicon (Si) is found naturally in soils as silica or silicate due to its strong affinity for oxygen. It is the second most abundant element in the Earth’s crust and soils, and its agronomic relevance has increased markedly in recent years [7,13,14,15]. Plants absorb silicon in the form of monosilicic acid [Si(OH)_4_] from the soil, and the plant’s roots translocate it to its various parts [16]. It is considered a “quasi-essential element” for plant growth due to its various beneficial roles in plant development and its ability to mitigate both abiotic and biotic stress [13,16]. Thus, a broad overview of the literature suggests that the application of silicon may play a significant role in mitigating stress toxicity in plants.

Among the various existing technologies, nanotechnology has emerged as a key tool in managing agricultural productivity. Nanotechnology in agriculture primarily focuses on the ecological production of nanomaterials for farming applications, including nanofertilizers, nanoencapsulates, nanofilms, and nanoparticles (NPs) [14,17,18].

The agricultural use of nanoparticles can enhance soil quality, improve plant–water relations, boost nutrient uptake and availability, promote growth and productivity, and protect crops from various biotic and abiotic stresses [19,20,21].

SiO_2_ nanoparticles (SiNPs) are among the most widely used in agriculture because they are generally inert and readily absorbed by plants [22,23]. Furthermore, continuous exposure of plants to nano-forms of silica in the soil suggests that they are non-toxic [22]. For instance, SiNP supplementation has been observed to enhance abiotic stress tolerance in crops such as tomato (*Solanum lycopersicum* L.) [14,22,24,25], wheat (*Triticum aestivum* L.) [7], rice (*Oryza sativa* L.) [26], maize (*Zea mays* L.) [27], rose (*Rosa hybrida* L.) [28], and pea (*Pisum sativum* L.) [16].

SiNPs have positive effects on plant growth [16,29,30], physiology [31], and protection of plants [26], as they can significantly enhance sugar metabolism [29], reactive oxygen species (ROS) metabolism [32], and water absorption and nutrient supply [33]. They also positively regulate photosynthesis and gas exchange [34,35] and activate metabolic processes, thereby improving the antioxidant defense system and nitrogen metabolism [16,36,37]. Another positive impact of the application of SiNPs is the mitigation of the adverse effects of different abiotic stresses, such as UV radiation [7], metal toxicity [14], saline stress [34], and biotic stress [38,39,40]. SiNPs can also be directly used as nano-pesticides, nano-herbicides, and nano-fertilizers [41,42].

The effects of silicon nanoparticles (SiNPs) in mitigating UV-B stress have been previously investigated by [7] in wheat (*Triticum aestivum* L.). However, the impact of SiNPs on other plants, including tomatoes, under UV-B stress remains unknown.

This study aims to enhance the resistance of tomato plants to UV-B radiation and alleviate UV-B-induced stress through the use of nanotechnology-based treatments with silica nanoparticles. To assess the effects, photosynthetic parameters, including stomatal water vapor conductance, net CO_2_ assimilation rate, transpiration rate, and intercellular CO_2_ molar fraction, as well as the content of photosynthetic pigments (chlorophylls and carotenoids) and monoterpenes, were evaluated in tomato plants in comparison to control groups after SiNP treatments and controlled UV-B exposure. Additionally, to assess the nutritional quality of the fruit, the total carotenoid and lycopene content in tomato fruits was also analyzed.

## 2. Results and Discussions

### 2.1. Characterization of SiNPs Material

**Figure 1:**
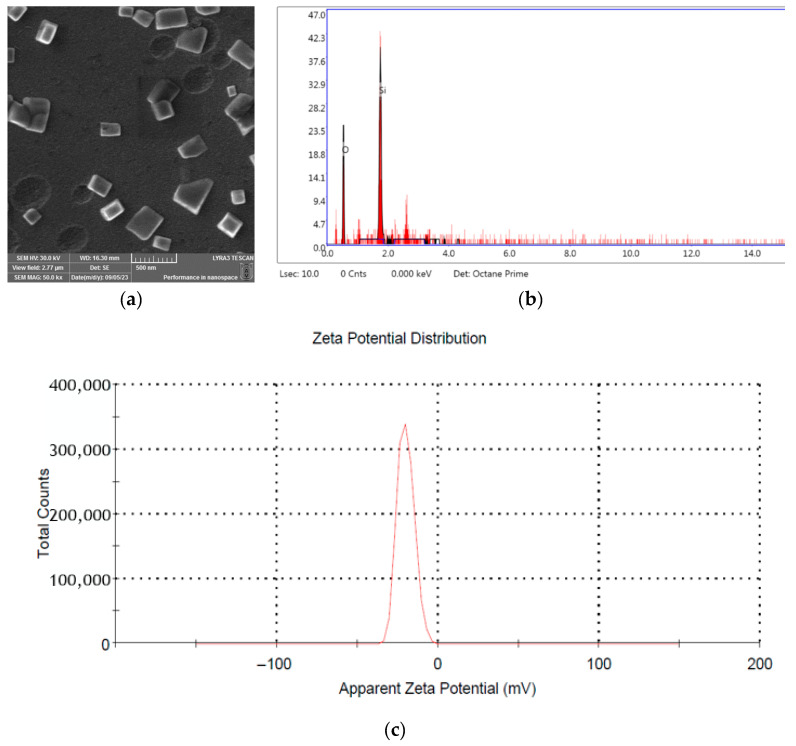
Characterization of silicon nanoparticles (SiNPs) by various techniques: SEM micrograph of silicon nanoparticles (**a**), EDS spectrum and elemental analysis of synthesized SiNPs (**b**), and zeta potential distribution (**c**).

As the primary objective of the present study was to develop a sustainable approach to UV-B protection in plants by stimulating their innate defense mechanisms, a straightforward method for synthesizing SiNPs was proposed based on a previously published method (as described in the Materials and Methods section). The resulting material was subjected to SEM analysis (Figure 1a), which revealed that the SiNPs have a crystalline cubic-shaped morphology. The SEM micrographs revealed nanoparticles with an average size of 178.76 ± 47.38 nm, presenting a slightly larger particle size compared to the DLS results. These differences may be attributed to particle agglomeration during sample storage and the drying step in SEM sample preparation, as opposed to DLS measurements performed on freshly prepared solutions containing SiNP samples. The EDS analysis (Figure 1c) showed a composition of 35.9 wt% Si and 64.1 wt% O, clearly indicating the absence of impurities in the material.

The dynamic light scattering data reveal a unimodal hydrodynamic diameter of 78.43 ± 1.6 nm. In contrast, the electrophoretic mobility measurements indicate an average ζ-potential of −20 ± 0.6 mV (Figure 1b). Empirical colloid stability guidelines categorize dispersions with |ζ| values ranging from 20 to 30 mV as “moderately stable.” In contrast, those with values exceeding ±30 mV are classified as “highly stable” [43]. Consequently, the observed surface charge is sufficient to produce electrostatic repulsion, which counteracts van der Waals attraction, thus minimizing particle–particle collisions and inhibiting aggregation. The observed magnitude of the ζ-potential, along with the narrow size distribution, is anticipated to preserve the physicochemical integrity of the nanodispersion for a minimum duration under ambient storage conditions.

### 2.2. Influence of SiNP Treatment on Photosynthetic Parameters in Tomato Plants

According to the proposed experimental model, the effects of SiNP treatment on photosynthetic parameters were assessed through foliar application of a SiNP solution (final concentration of 5.2 × 10^−5^ nM) on tomato plants after seven weeks of growth (vegetative growth stage, ensuring a sufficient leaf surface for application and parameter measurement).

**Figure 2:**
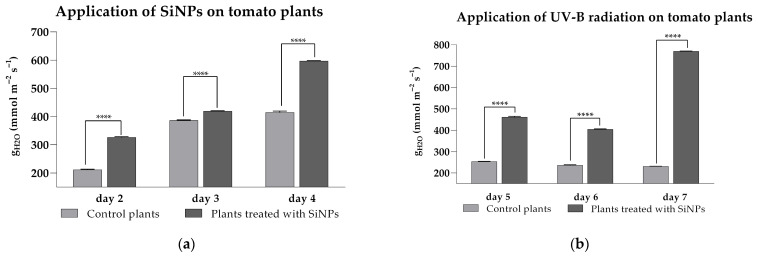
Stomatal conductance of tomato leaves after SiNP foliar application under ambient (**a**) and enhanced (**b**) UV-B radiation. Ordinary one-way ANOVA: Bonferroni’s multiple comparison test for emphasizing statistical differences (*p* < 0.0001, extremely significant ****; *p* = 0.0001 to 0.001, extremely significant ***; *p* = 0.001 to 0.01, very significant **; *p* = 0.01 to 0.05, significant *; *p* > 0.05, insignificant ns).

Across the three-day measurement period, foliar application of SiNPs elicited a highly significant increase in stomatal conductance (*p* < 0.0001) relative to controls under ambient UV-B (Figure 2a), with values of 326 ± 2.7 to 596 ± 1.5 mmol m^−2^ s^−1^ in treated leaves versus 211 ± 2.1 to 414 ± 4.4 m^−2^ s^−1^ in controls. Under enhanced UV-B (Figure 2b), control plants maintained low conductance (230 ± 0.5–252 ± 2.0 m^−2^ s^−1^), whereas SiNP-treated plants exhibited substantially higher values (461 ± 3.4–769 ± 0.8 mmol m^−2^ s^−1^). Taken together, these results indicate that SiNP treatment promotes wider stomatal opening and preserves gas exchange capacity under both ambient and elevated UV-B.

**Figure 3:**
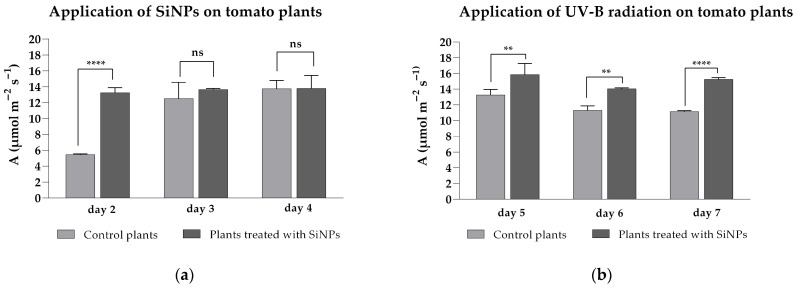
CO_2_ net assimilation rates of tomato leaves after SiNP foliar application under ambient (**a**) and enhanced (**b**) UV-B radiation. Ordinary one-way ANOVA: Bonferroni’s multiple comparison test for emphasizing statistical differences (*p* < 0.0001, extremely significant ****; *p* = 0.0001 to 0.001, extremely significant ***; *p* = 0.001 to 0.01, very significant **; *p* = 0.01 to 0.05, significant *; *p* > 0.05, insignificant ns).

Based on the measured net CO_2_ assimilation rates, it was observed that treated plants showed a significantly higher assimilation rate only on the first day after treatment, with a value of 13.26 ± 0.63 μmol m^−2^ s^−1^ compared to 5.44 ± 0.09 μmol m^−2^ s^−1^ in the untreated group. In contrast, similar values were recorded on the second (ns, *p* = 0.7749) and third (ns, *p* > 0.9999) days of treatment for both treated and untreated plants (Figure 3a), with A values ranging from 12.50 ± 2.04 to 13.79 ± 1.63 μmol m^−2^ s^−1^. After the application of enhanced UV-B radiation, significantly higher values were obtained for the treated plants during the three days of exposure (Figure 3b), with declining A values between 11.12 ± 0.14–13.26 ± 0.70 μmol m^−2^ s^−1^ for control plants. After UV-B exposure, the SiNP-treated plants showed A values between 14.03 ± 0.11 and 15.84 ± 1.41 μmol m^−2^ s^−1^, which were similar to or higher than those obtained during the three days of foliar application (days 2–4).

**Figure 4:**
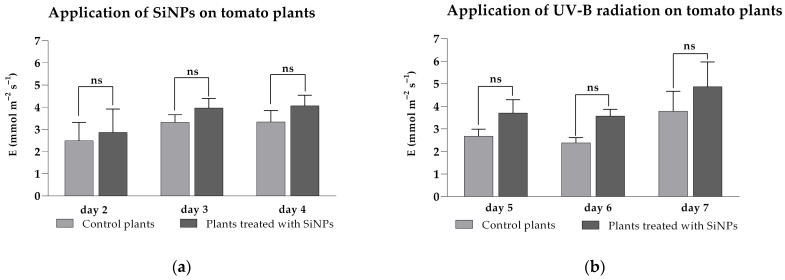
Transpiration rates of tomato plant leaves after SiNP foliar application under ambient (**a**) and enhanced (**b**) UV-B radiation. Ordinary one-way ANOVA: Bonferroni’s multiple comparison test for emphasizing statistical differences (*p* < 0.0001, extremely significant ****; *p* = 0.0001 to 0.001, extremely significant ***; *p* = 0.001 to 0.01, very significant **; *p* = 0.01 to 0.05, significant *; *p* > 0.05, insignificant ns).

Transpiration rate is another parameter that provides relevant information regarding photosynthesis, as it is directly correlated with stomatal conductance. A higher stomatal conductance results in a higher transpiration rate in leaves. Based on the measured data, it was observed that under ambient UV-B radiation, the treated plants showed an increasing trend in transpiration rates after treatment was performed (Figure 4a) when compared to untreated plants (2.86 ± 1.04 to 4.05 ± 0.48 mmol m^−2^ s^−1^ for treated plants, from 2.48 ± 0.82 to 3.33 ± 0.51 mmol m^−2^ s^−1^ for untreated plants). A similar increase was recorded during UV-B exposure for treated plants when compared to the untreated group (Figure 4b), with values for E ranging from 3.69 ± 0.59 to 4.86 ± 1.09 mmol m^−2^ s^−1^ for treated plants and from 2.37 ± 0.23 to 3.78 ± 0.88 mmol m^−2^ s^−1^ for untreated plants. Despite the observed increasing tendency, statistical analysis showed no significant differences between the groups during the 3 days of SiNP treatment (*p* > 0.9999 for day 2, *p* = 0.7633 for day 3, *p* = 0.5977 for day 4) or the 3 days of UV-B exposure (*p* = 0.2361 for day 5, *p* = 0.1368 for day 6, *p* = 0.1932 for day 7).

**Figure 5:**
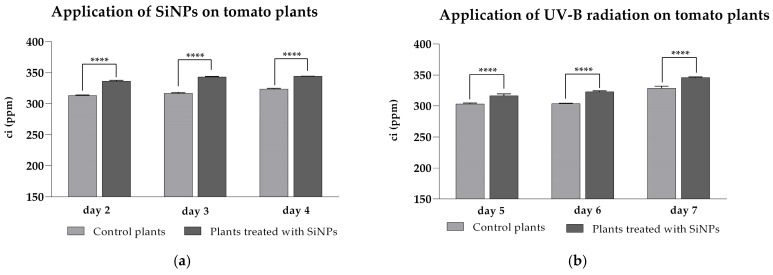
Intercellular CO_2_ molar fraction of tomato leaves after SiNPs foliar application under ambient (**a**) and enhanced (**b**) UV-B radiation. Ordinary one-way ANOVA: Bonferroni’s multiple comparison test for emphasizing statistical differences (*p* < 0.0001, extremely significant ****; *p* = 0.0001 to 0.001, extremely significant ***; *p* = 0.001 to 0.01, very significant **; *p* = 0.01 to 0.05, significant *; *p* > 0.05, insignificant ns).

The intercellular CO_2_ molar fraction (Ci) directly correlates with the amount of carbon dioxide that can be fixed in the intercellular space of the plant’s cells and is taken up by chloroplasts for carbon fixation and biomass generation. The Ci concentration directly affects the first step of carbon fixation in the Calvin cycle, as the activity of the Rubisco enzyme depends on the available CO_2_ in the intercellular space. As shown in Figure 5a, under ambient UV-B conditions, treated plants exhibited significantly higher Ci values (ranging from 336 ± 1.3 to 344 ± 0.1 ppm) compared to untreated plants (313 ± 0.9 to 323 ± 1.1 ppm).

Elevated UV-B exposure reduced intercellular CO_2_ concentration (Ci) in all tomato plants (Figure 5b). However, foliage treated with silicon nanoparticles (SiNPs) retained significantly higher Ci values—ranging from 316 ± 2.9 to 345 ± 1.0 ppm—than the untreated controls (*p* < 0.05). These data indicate that foliar SiNP application mitigates the UV-B-induced decline in internal CO_2_ availability, thereby helping sustain the carbon supply required for photosynthetic assimilation under stress conditions.

Taking into account all photosynthetic parameters measured for tomato plants treated with SiNPs, a synergistic effect was observed, especially under enhanced UV-B radiation. The stomatal conductance values of leaves were found to be much higher when SiNPs were applied to the foliage without any external stress factors, suggesting an increased number of opened stomata on the epidermis of plant leaves, favoring transpiration and increasing gas exchange [44]. In contrast, enhanced UV-B exposure leads to much lower values for the untreated plants than those obtained for the SiNP-treated ones. Considering that UV-B can indirectly affect photosynthetic parameters by reducing stomatal conductance, altering leaf anatomy, and increasing leaf thickness [45], a clear positive influence on stomatal conductance is observed in the treatment with SiNPs, suggesting that the presence of silica nanoparticles could potentially enhance stomatal structure and function. Silicon has also been shown to improve plant resistance to drought stress in sugarcane leaves by directly affecting stomata ultrastructure and improving photosynthetic efficiency [46]. Building on the SiNP-induced enhancement of stomatal conductance (GH_2_O), treated leaves also sustained significantly higher transpiration (E) and net CO_2_ assimilation (A) after UV-B exposure, which, in turn, maintained a larger intercellular CO_2_ mole fraction (Ci) relative to the untreated controls. Silicon can deposit in guard-cell walls, enhancing K^+^-mediated osmotic regulation of the aperture and thereby keeping stomata functionally open under stress [47]. In our experimental regime, the UV-B treatment was sufficient to elicit precise physiological and metabolic adjustments in both control and SiNP-treated plants (see preceding and subsequent). The absence of a statistically significant treatment effect on this trait is therefore unlikely to reflect a lack of UV-B responsiveness per se. Instead, it may indicate a preferential shift toward photoprotective metabolism—accumulation of flavonoids and other phenylpropanoids that function as UV screens and antioxidants—over changes in hydraulic conductance or aquaporin-mediated water transport. Such reallocations are well documented for UV-B acclimation via the UVR8–HY5 pathway and result in heightened levels of flavonols and related polyphenolics that stabilize photosynthetic performance without necessarily altering transpiration at the whole-leaf scale [48]. Within this metabolic context, the primary effect of SiNPs in our study appears to be the preservation of stomatal function and carbon assimilation rather than a detectable enhancement of leaf-level water fluxes. Future work quantifying total phenolics/flavonoids and profiling aquaporin expression will be necessary to disentangle these mechanisms [49]. Comparable increases in G_H2O_, E, and A—and, consequently, improved photosynthetic performance—have been reported in other crops supplied with bulk or nano-Si formulations [45], underscoring the generality of the protective effect. Alam et al. have also demonstrated that SiNP treatment of *Solanum lycopersicum* leads to a substantial increase in net photosynthetic rate for plants grown with and without salt stress as an abiotic stress factor [50]. Furthermore, they also demonstrated that SiNPs enhance the activity of cellular enzymes, such as Rubisco, thereby favoring CO_2_ fixation and subsequent conversion into sugars. Therefore, the current data support the beneficial effects of the foliar application of SiNPs on photosynthetic parameters in tomato plants while also reducing the amount of damage caused by enhanced UV-B exposure. To contextualize these responses, several complementary mechanisms may explain how foliar SiNPs interact with stomatal regulation under UV-B. First, silicon can accumulate in guard-cell walls and subtly modify their mechanics and ion fluxes, supporting greater aperture by facilitating K^+^-driven turgor maintenance. Second, Si-mediated attenuation of oxidative stress can dampen abscisic acid (ABA) signaling cascades that otherwise promote UV-B-associated stomatal closure, thereby preventing unnecessary loss of conductance under moderate stress. Third, reports of Si-induced up-regulation of plasma-membrane intrinsic protein (PIP) aquaporins and improved leaf/root hydraulic conductance provide a hydraulic basis for stabilizing guard-cell turgor and sustaining gas exchange. Finally, enhancements in chloroplast photochemistry (e.g., preserved PSII performance and Rubisco activity) reduce feedback limitations on CO_2_ diffusion, indirectly favoring higher intercellular CO_2_ and net assimilation at a given vapor pressure deficit. Because foliar nano-Si can access tissues via stomata and trichomes, the primary site of action is plausibly the epidermis and the adjacent palisade mesophyll [51,52].

### 2.3. Influence of SiNP Treatment on Targeted Photosynthesis-Related Secondary Metabolites

#### 2.3.1. Analysis of BVOCs Emitted for SiNP-Treated and -Untreated Tomato Plants

**Figure 6:**
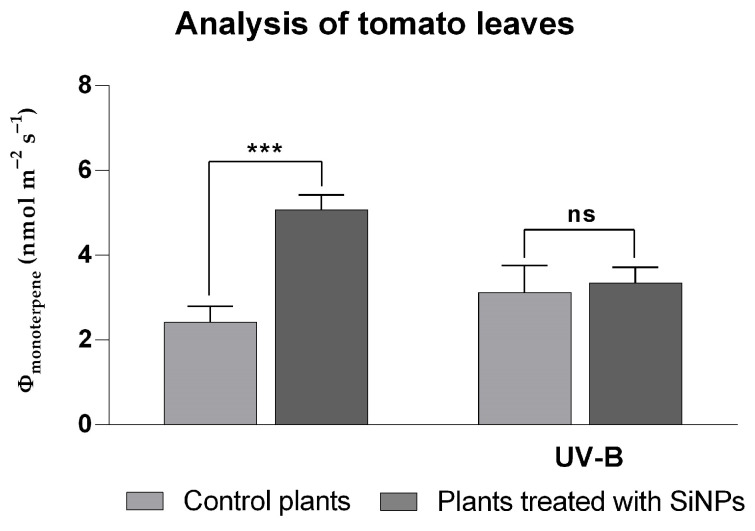
Total monoterpene emissions after SiNP foliar application and after exposure to enhanced UV-B radiation. Ordinary one-way ANOVA: Bonferroni’s multiple comparison test for emphasizing statistical differences (*p* < 0.0001, extremely significant ****; *p* = 0.0001 to 0.001, extremely significant ***; *p* = 0.001 to 0.01, very significant **; *p* = 0.01 to 0.05, significant *; *p* > 0.05, insignificant ns).

Biogenic volatile organic compounds (BVOCs) have been widely recognized as important indicators linking various biotic and abiotic stress factors with plant metabolic responses [53,54], including in tomato plants [55]. In the current study, monoterpenes emitted by the plant’s leaves were analyzed directly from the plants after three days of SiNP treatment (on the fourth day) and after three days of exposure to enhanced UV-B radiation. The major monoterpenes identified in the tested tomato variety were α-thujene, β-pinene, 3-carene, isothujol, and carvacrol. In addition to monoterpenes, several sesquiterpenes were also detected, including longifolene and isogermacrene. The obtained data (presented in Figure 6) revealed that the total amount of monoterpenes increased slightly from 2.42 ± 0.36 to 5.07 ± 0.34 nmol·m^−2^ s^−1^ after treatment with nanoparticles, causing a slight stress for the plants, as expected. However, during enhanced UV-B exposure, the differences between the control group (3.11 ± 0.63 nmol·m^−2^ s^−1^) and the treated group (3.34 ± 0.36 nmol m^−2^ s^−1^) were not found to be significant, with similar levels of monoterpene emissions. Although the literature on BVOC emissions of tomato plants under abiotic stress is scarce, the slightly higher emission of monoterpenes observed after SiNP treatment under ambient radiation follows other reported data that showed an increase in secondary metabolite production and BVOC emissions when nanoparticles were applied to various plants [56]. In line with the adjacent endpoints, the UV-B regime applied here was sufficient to elicit physiological and metabolic acclimation in both control and SiNP-treated plants. The absence of a statistically significant effect on this particular trait, therefore, likely reflects response prioritization rather than inadequate stimulus. Early UV-B perception via the UVR8 photoreceptor occurs within minutes and rapidly drives HY5-dependent transcriptional programs; among the most consistent outputs is the up-regulation of phenylpropanoid/flavonoid biosynthesis that enhances UV screening and antioxidant capacity. Such preferential allocation to photoprotective metabolites can stabilize photosynthetic function without producing measurable changes in the trait assessed under short exposures. This interpretation aligns with current mechanistic and crop-oriented reviews documenting rapid UVR8–HY5 signaling and the prominence of flavonoid-based acclimation under UV-B [57]. For example, in tomato leaves, a 4 h UV-B treatment failed to enhance the emission of monoterpene biogenic volatile organic compounds (BVOCs), whereas extending the exposure to 8–12 h produced a pronounced post-irradiation surge in monoterpene release [58]. The temporal lag likely indicates the duration required for the transcriptional activation of the pertinent terpene-synthase genes and the subsequent accumulation of their products, highlighting the significance of exposure duration in evaluating UV-B responses.

#### 2.3.2. Analysis of Photosynthetic Pigments for SiNP-Treated and -Untreated Tomato Plants

**Figure 7:**
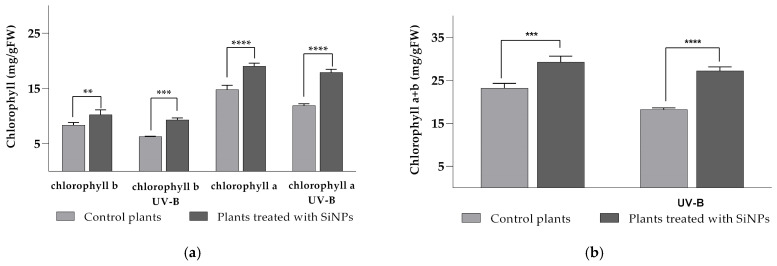
The chlorophyll *a* and *b* content (**a**) and total amount of chlorophyll (*a* + *b*) (**b**) in tomato leaves after SiNP foliar application and after enhanced UV-B radiation exposure. Ordinary one-way ANOVA: Bonferroni’s multiple comparison test for emphasizing statistical differences (*p* < 0.0001, extremely significant ****; *p* = 0.0001 to 0.001, extremely significant ***; *p* = 0.001 to 0.01, very significant **; *p* = 0.01 to 0.05, significant *; *p* > 0.05, insignificant ns).

As primary photosynthetic pigments in plants, the chlorophyll levels in plant leaves directly correlate with their photosynthetic activity. Chlorophylls (*a*, *b*, and total) levels in leaves of tomato plants treated with SiNPs and exposed to enhanced UV-B radiation showed statistical differences when compared to untreated plants (Figure 7). Both chlorophyll *a* and *b* contents increased by 22.4% and 47.9% after treatment with SiNPs compared to untreated plants (Figure 7a). After enhanced UV-B radiation treatment was applied, chlorophyll content slightly decreased in both treated and untreated plants, but the SiNP-treated group maintained an increased value by 28.5% in chlorophyll *a* and 50.4% in chlorophyll *b* when compared to the untreated plants. Similarly, the total amount of chlorophylls was significantly higher compared to the control group (Figure 7b), with increases of 26.3% after SiNP application and 49.5% after UV-B exposure. Comparing control groups, a significant decrease in chlorophyll levels was observed after plants were exposed to enhanced UV-B due to the rupture of chloroplasts and subsequent chlorophyll degradation. Fortunately, the negative effect was mitigated by the application of SiNPs, leading to higher levels of both individual and total chlorophyll content. These observations may be explained by the uptake of SiNPs in the leaf epidermis, which stimulates chlorophyll synthesis [23], or by the deposition of silica in the epidermal cell wall, providing mechanical protection against UV stress [59,60]. Pinedo-Guerrero et al. also demonstrated that SiNP treatment of tomato plants led to a significant increase in chlorophyll levels under both ambient and salt stress conditions [61]. Available data from the literature also show that supplementation with silicon or silicon nanoparticles, either through soil or foliar application, resulted in a significant increase in chlorophyll levels in the leaves of *Zea mays*, *Triticum aestivum*, *Saccharum officinarum*, *Hordeum vulgare,* and other economically relevant crops [23]. Our analysis does not clarify whether the SiNP-associated increase in chlorophyll indicates enhanced biosynthesis or decreased degradation; both possibilities are feasible. Si/SiNPs have been shown to support tetrapyrrole production on the biosynthetic side by up-regulating important genes, including HEMA1, CHLH/GUN5, and POR. This helps make chlorophyll from scratch [62]. On the catabolic side, Si can slow down the PAO/phyllobilin degradation pathway by lowering the activity and/or expression of chlorophyll catabolic genes (e.g., SGR/NYE1, PPH, PAO) [63].

**Figure 8:**
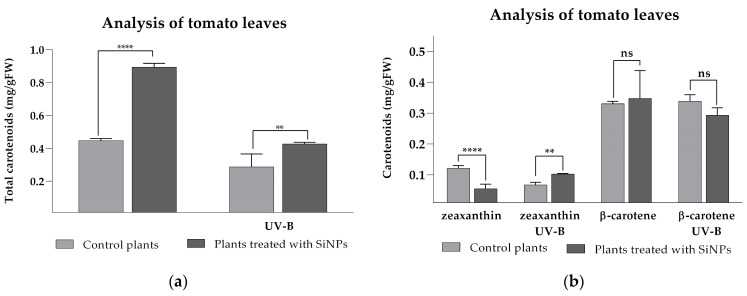
The total carotenoid content (**a**) and amount of individual major carotenoids zeaxanthin and β-carotene (**b**) in tomato leaves after SiNP foliar application and after enhanced UV-B radiation exposure. Ordinary one-way ANOVA: Bonferroni’s multiple comparison test for emphasizing statistical differences (*p* < 0.0001, extremely significant ****; *p* = 0.0001 to 0.001, extremely significant ***; *p* = 0.001 to 0.01, very significant **; *p* = 0.01 to 0.05, significant *; *p* > 0.05, insignificant ns).

As secondary photosynthetic pigments in leaves, carotenoid pigments play a crucial role in mitigating the harmful effects of enhanced UV-B damage and have a prominent function in protecting the photosynthesis apparatus from damage under high light conditions [64]. Their antioxidant properties help mitigate the harmful effects of UV-B-induced reactive oxygen species (ROS), either by quenching photosensitized triplet chlorophyll or inactivating superoxide anion radicals (O_2_•^−^) [65]. Similarly to measured chlorophyll levels, total carotenoid levels in tomato leaves were also enhanced significantly after SiNP treatment was applied, with an increase of 100.5% when compared to the control (Figure 8a). Enhanced UV-B treatment resulted in lower amounts of carotenoids for both treated and untreated plants; however, the treated plants still showed an increase of 48.5% in total carotenoids than the control. Analysis of individual carotenoids (Figure 8b), specifically β-carotene and zeaxanthin, revealed that β-carotene levels did not show statistical differences between the treated and untreated groups (ns, *p* = 0.7568), even after UV-B exposure (ns, *p* = 0.0713). In contrast, zeaxanthin levels showed a significant decrease after SiNP treatment but a significant increase after UV-B exposure. The divergence between the total carotenoid pool and the stable concentrations of zeaxanthin and β-carotene can be explained by a preferential enrichment of other xanthophylls—principally lutein, violaxanthin, and neoxanthin—which collectively raise the bulk carotenoid estimate while leaving the two quantified pigments unchanged. A similar pattern has been observed in tomatoes, where supplementation with silicon nanoparticles (SiNPs) under pathogen challenge resulted in a broad increase in carotenoid accumulation [66]. The capacity of SiNPs to preserve or even enhance carotenoid biosynthesis appears to be general; significant elevations in total or specific carotenoids have also been reported for strawberries, wheat, and potatoes grown under diverse abiotic stresses [67,68]. Collectively, these findings suggest that SiNPs help sustain the photoprotective and antioxidant functions of carotenoids by up-regulating multiple branches of the carotenoid biosynthetic pathway rather than selectively boosting individual pigments.

#### 2.3.3. Analysis of Lycopene and Total Carotenoid Content in Tomato Fruit from SiNP-Treated and -Untreated Plants

**Figure 9:**
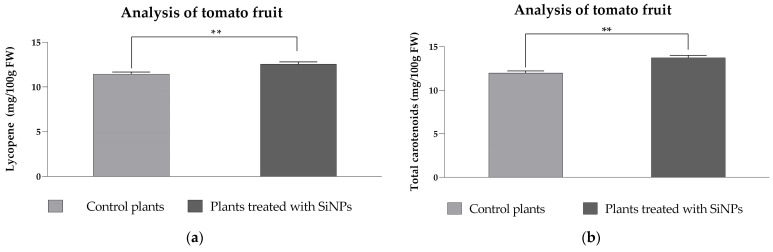
The lycopene content (**a**) and total amount of carotenoids (**b**) in tomato fruit after SiNP foliar application and enhanced UV-B radiation exposure. Ordinary one-way ANOVA: Bonferroni’s multiple comparison test for emphasizing statistical differences (*p* < 0.0001, extremely significant ****; *p* = 0.0001 to 0.001, extremely significant ***; *p* = 0.001 to 0.01, very significant **; *p* = 0.01 to 0.05, significant *; *p* > 0.05, insignificant ns).

Secondary metabolites in plants, such as carotenoids, are closely linked to human health due to their nutritional and antioxidant properties. However, there is limited data reported on the effect of SiNP treatment on secondary metabolite levels in tomato fruits. Therefore, the total carotenoid and lycopene contents were evaluated in ripened tomato fruit obtained from plants grown with and without SiNP treatment after exposure to enhanced UV-B radiation. The tomato plants were planted and allowed to grow under ambient conditions, and the tomato fruits were harvested at full maturity. The results revealed that the amount of lycopene did not show a major difference within tomatoes from the control and treated plants; however, a significant increase of 9.8% for tomatoes harvested from the SiNP-treated group (*p* = 0.0050) was observed (Figure 9a). Similarly, the total carotenoid content also showed a significant increase of 14.6% for the SiNP-treated plants with regards to the untreated plants (*p* = 0.0012) (Figure 9b). The obtained data suggest that the investigated SiNP treatment not only does not negatively impact the growth or nutritional values of tomato fruit but also slightly improves the nutrient content, particularly in terms of carotenoid content. However, other reports on the lycopene and β-carotene concentrations in tomato fruit treated with silicon (Si) did not find significant differences between treated and untreated plants under normal growth conditions [69]. A more recent study demonstrated that foliar application of both K_2_SiO_3_ and SiO_2_ nanoparticles to tomato plants resulted in a significantly higher concentration of β-carotene in harvested tomato fruit [61].

In contrast, no significant differences were observed in lycopene content compared to control plants [61]. González-Moscoso et al. also noted that SiNP treatment of tomato plants led to a significantly higher content of lycopene and β-carotene in tomato fruit; however, the treatment period was longer and with a higher concentration of SiNPs [70]. Taken together, it can be presumed that the influence of SiNPs on the carotenoid content of tomato fruit grown under enhanced UV-B stress is likely dependent on the type and concentration of nanoparticles applied, as well as the treatment period. The weaker carotenoid gain in the fruit relative to leaves likely reflects both distribution and regulation. After foliar application, Si/SiO_2_ nanoparticles tend to deposit locally in leaf epidermis and show limited long-distance translocation, so less silicon reaches developing fruit.

Meanwhile, leaf carotenoids respond rapidly to UV-B with photoprotective xanthophyll enrichment. In contrast, fruit lycopene is controlled by ripening-specific programs and chromoplast development, which are less sensitive to short-term SiNP/UV-B cues. UV-B timing and dose also matter; regimes that alter leaves may be insufficient to shift fruit lycopene. Targeted follow-ups—quantifying Si in leaves vs. fruit and profiling ripening/carotenoid genes—would clarify whether limited translocation or metabolic control is the dominant constraint [71].

### 2.4. Correlations Between Physiological and Biochemical Parameters in Tomato Plants

**Figure 10:**
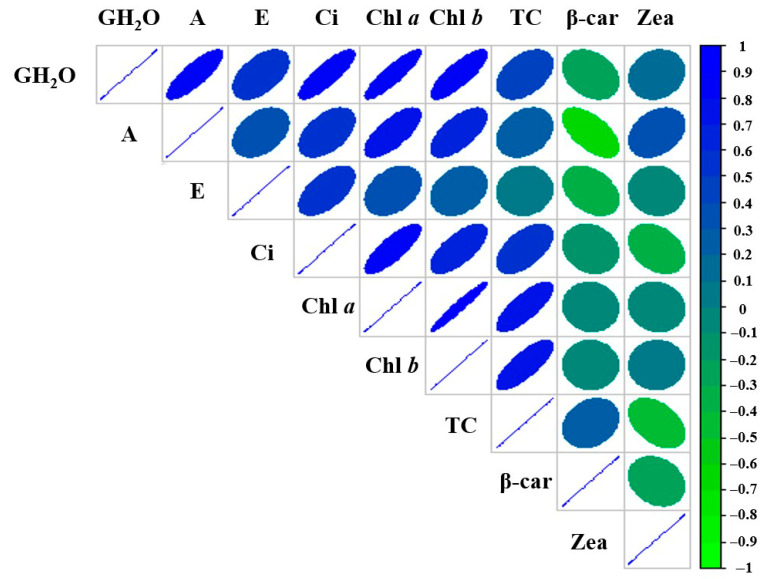
Correlogram of physiological and biochemical parameters in tomato plants. The diagram shows Pearson’s correlation coefficients, with blue tones indicating positive correlations and green tones negative correlations. The color intensity and the narrowness of the ellipses reflect the correlation strength (scale from −1 to +1). Abbreviations: GH_2_O = stomatal conductance to water vapor; A = net CO_2_ assimilation rate; E = transpiration rate; Ci = intercellular CO_2_ molar fraction; Chl *a* = chlorophyll *a*; Chl *b* = chlorophyll *b*; TC = total carotenoids; β-car = β-carotene; Zea = zeaxanthin.

A statistical analysis of the interactions among physiological and biochemical parameters in tomato plants subjected to UV-B stress and silicon nanoparticle treatment was conducted by examining the relationships between variables (Figure 10). The analysis revealed strong positive correlations between photosynthetic parameters, including net CO_2_ assimilation rate (A), stomatal conductance to water vapor (GH_2_O), transpiration rate (E), and chlorophyll *a* and *b* (Chl *a*, Chl *b*), suggesting that photosynthetic activity was closely linked to stomatal regulation and pigment levels. Under light stress, plants have evolved protective mechanisms to preserve photosynthetic efficiency, which include the accumulation of pigments, including carotenoids [72,73]. Consistent with this, the present study revealed significant positive correlations between chlorophylls and total carotenoids (TC), β-carotene (β-car), and zeaxanthin (Zea). Moreover, zeaxanthin and β-carotene showed moderate negative correlations with GH_2_O, which may reflect the activation of the xanthophyll cycle as a protective strategy under conditions of stomatal limitation or increased oxidative stress [74]. On the other hand, β-carotene exhibited weaker correlations with photosynthetic parameters compared to other pigments. This observation may be attributed to its predominantly structural and photoprotective role rather than a dynamic involvement in photosynthetic regulation. In photosystem II (PSII), where stress-related damage is more pronounced under conditions such as UV-B exposure, β-carotene contributes primarily to the stabilization of pigment–protein complexes and the quenching of reactive oxygen species rather than directly participating in light harvesting. Unlike in photosystem I (PSI), where β-carotene has been shown to play a more active role in energy transfer, its function in PSII is mainly protective [75]. Altogether, these correlations indicate a coordinated physiological and biochemical response, where pigment composition interacts with stomatal behavior to modulate together the photosynthetic function under UV-B stress.

## 3. Materials and Methods

### 3.1. Plant Material and Growth Conditions

Tomato (*Solanum lycopersicum* L.) seeds of the semi-determinate Final F1 variety were planted in the greenhouse of the University of Agricultural Sciences and Veterinary Medicine Cluj-Napoca. In the fifth true leaf stage, seedlings were transplanted into 12 L pots on a peat substrate. Seven weeks after transplanting, the resulting tomato plants were divided into two groups: a control group and an experimental group. The experiment started at this point, allowing the plants sufficient time to grow and ensuring that any changes in photosynthetic activity at the leaf blade level could be effectively analyzed.

### 3.2. Chemical Reagents

All chemicals and reagents used in the study were of analytical grade or HPLC grade and were purchased from Sigma Aldrich or Merck unless otherwise stated.

### 3.3. Synthesis and Characterization of Silicon Nanoparticles (SiNPs)

The synthesis and characterization of silicon nanoparticles (SiNPs) were carried out using a method similar to the one described by Tripathi et al. [7], with slight modifications. In this process, sodium silicate (Na_2_SiO_3_) was employed in a sol–gel method. Initially, Na_2_SiO_3_ was dissolved in 475 mL of distilled water (5 mM), and the pH was adjusted to 3.0 using 1 mM HCl. The solution was then heated and stirred in a water bath at 75 °C for 2 h. The pH was raised to 7.0 by adding NH_4_OH, and the mixture was further stirred for 1.5 h at the same temperature. The Si solution was dialyzed using a dialysis bag (10k MWCO) to eliminate impurities such as Na^+^ and NH_4_^+^ ions. After dialysis, the obtained solution containing the nanoparticles was further used for SiNP analysis and plant treatment.

The characterization of SiNPs was conducted by using SEM-EDS (scanning electron microscopy with energy dispersive X-ray spectroscopy) analysis and zetasizer. The morphology and particle size of the SiNPs were analyzed using a scanning electron microscope (SEM; LYRA 3 XMU, Tescan, Czech Republic) operating at 30 kV. Several drops of the solution were applied to a stub covered with carbon tape, and images were captured at a magnification of 50,000×. Elemental analysis was conducted using an Energy Dispersive X-ray (EDX) spectroscope (EDAX Inc., Mahwah, NJ, USA). The mean size, polydispersity index (PDI), and zeta potential of the nanoemulsions were determined using the dynamic light scattering (DLS) method with a Zetasizer Nano ZS analyzer (Malvern Instruments Worcestershire, UK). Measurements were performed at 25 °C using a Doppler electrophoresis laser with a wavelength of 633 nm. The optical parameters were set according to the Mie theory, with a refractive index of 1.48 and an absorption index of 0.01. Measurements were performed in triplicate at 25 °C. The concentration and hydrodynamic size distribution of SiNPs were determined using Nanoparticle Tracking Analysis (NTA) on a NanoSight NS400 instrument (Malvern Panalytical, UK).

### 3.4. Experimental Model: SiNP Treatments and UV-B Exposure

After seven weeks of growth, a foliar application of a SiNP solution was performed on the experimental tomato plants (six plants for the control and six plants for the foliar application of SiNPs) to investigate the potential stimulation of the plant’s natural protection mechanisms. Approximately 400 mL of the SiNP solution was applied daily using a sprayer at 24 h intervals for three consecutive days. Plants that were not exposed to SiNP treatment were considered the control group. Control plants were sprayed with distilled water using the same sprayer, volume, timing, and procedure to account for any physiological responses to foliar wetting and handling. To avoid drift or cross-contamination, treatment groups were spatially separated during application and for 30 min thereafter. All physiological measurements were conducted only after leaves were visibly dry. After the SiNP treatment, all plants were exposed to UV-B radiation for 2 h/day for the next three days. The UV-B irradiance at the top of the plants under the tube was measured using a UV meter (PCE Instruments, Model PCE-UV34, Meschede, Germany). UV-B radiation was provided by an artificial source, with the plants placed under a 100 W UV-emitting fluorescent tube.

Meanwhile, UV-A and UV-C radiation were inhibited by covering the tubes with a cellulose diacetate film. The intensity of the applied radiation was 1.5 W/m^2^, in accordance with the relevant literature [3,76]. Ambient UV exposure had a mean value of 0,66 mW/cm^2^. Photosynthetic parameters were measured after each treatment, and secondary metabolites were measured after the final treatment with SiNPs. Additionally, photosynthetic parameters and secondary metabolites were measured following each UV-B exposure.

### 3.5. Measurement of Photosynthetic Parameters

Photosynthetic parameters of plants were monitored using the GFS 3000 Portable Gas Exchange System (Walz GmbH, Effeltrich, Germany) (Figure S1). This system features an environmentally controlled cuvette with an 8 cm^2^ window area and is equipped with a full-window leaf chamber with sample illumination. Measurements were conducted at a cuvette CO_2_ concentration of 400 μmol mol^−1^, a photosynthetic photon flux density of 1000 μmol m^−2^ s^−1^, a leaf temperature of 25 °C, and a chamber relative humidity of 65%. The airflow rate was maintained at 750 μmol s^−1^. Leaves were positioned side by side to enclose the gas exchange cuvette window fully and were stabilized until steady-state values of stomatal conductance to water vapor (g_H2O_), net CO_2_ assimilation rate (A), transpiration rate (E), and intercellular CO_2_ molar fraction (Ci) were achieved. These parameters were calculated based on these steady-state measurements, following the methodology described by von Caemmerer and Farquhar [77].

### 3.6. Measurement of Secondary Metabolites in Tomato Leaves

#### 3.6.1. BVOC Sampling and GC-MS Analysis

Biogenic volatile organic compound (BVOC) sampling was conducted daily from the outlets of each cuvette using adsorbent cartridges at a flow rate of 200 mL min^−1^ for 20 min. A constant-flow air sampling pump equipped with four ports (1003-SKC, SKC Inc., Houston, TX, USA) was used for this purpose. To account for background BVOC concentrations, additional samples were collected from the air inlet before the cuvettes. The adsorbent cartridges, designed to optimize the capture of plant volatiles ranging from C5 to C15 were packed with various grades of Carbopack, following protocols outlined in previous studies [78,79]. Analysis of the adsorbent cartridges focused on monoterpenes (α-thujene, β-pinene, 3-carene, isothujol, and carvacrol) and sesquiterpenes (longifolene and isogermacrene). This was performed using a Shimadzu TD20 automated cartridge desorber coupled with a Shimadzu 2010Plus GC-MS system (Shimadzu Corporation, Kyoto, Japan) following previously established GC-MS methods [78,79]. Compound identification and quantification were achieved using authentic standards (Sigma-Aldrich, Taufkirchen, Germany). Calculations of volatile emission rates were performed by subtracting background (blank) emissions from those of the leaf samples. Total BVOCs were calculated as the sum of all identified individual compounds and expressed as nmol m^−2^ s^−1^.

#### 3.6.2. Chromatographic Analysis of Photosynthetic Pigments

Leaf samples (8 cm^2^) were collected after gas exchange measurements and immediately frozen in liquid nitrogen. The samples were weighed and then ground in liquid nitrogen using a mortar and pestle. The pigments were extracted in ice-cold 70% acetone. The extract was centrifuged using a Hettich 320R Universal centrifuge (Hettich GmbH, Tuttlingen, Germany) at 0 °C and 9500× *g* for 3 min, and the supernatant was collected. The extraction was repeated at least three times with small volumes of acetone until the supernatant became colorless. The supernatant fractions were pooled, brought to a final volume of 1 mL with acetone, and filtered through a 0.45 μm PTFE membrane filter (VWR International, Radnor, PA, USA).

The extracts were analyzed for carotenoids and chlorophylls using a UHPLC (NEXERA 8030, Shimadzu, Japan) equipped with a diode array detector (DAD), following a modified method from Opriş et al. [80]. A Nucleosil 100-3 C18 reversed-phase column (4.0 mm i.d. 150 mm column length, 3 µm particle size, Macherey–Nagel, Düren, Nordrhein-Westfalen, Germany) was used for the separation. The column temperature was maintained at 10 °C, and the flow rate was set at 1.5 mL min^−1^. The mobile phases consisted of buffered ultra-pure water (0.1 M sodium phosphate buffer, pH = 8) (Solvent A) and HPLC-grade acetone (Solvent B). The elution program was as follows: 25% (A) and 75% (B) for the first 7.5 min, followed by a 9.5 min linear gradient to 100% (B), which was maintained for 3 min. The mobile phase was then returned to the initial composition (25% A and 75% B) via a 2 min linear gradient. Flow rate and column temperature were kept constant throughout. The HPLC system was calibrated using commercially available pigments, including chlorophyll a, chlorophyll b, β-carotene, zeaxanthin, and lutein (Fluka, Steinheim, Germany). The wavelengths used for absorbance maxima were 430 nm for chlorophyll a, 450 nm for neoxanthin and violaxanthin, and 455 nm for the remaining pigments (chlorophyll b, β-carotene, zeaxanthin, and lutein). The results were expressed as milligrams per gram of fresh weight (FW).

#### 3.6.3. Total Carotenoid and Lycopene Concentrations in Tomato Fruits

Tomato fruits were harvested approximately 2 months after the final treatment application of SiNPs (Figure S2). Carotenoids were extracted from 1 g of homogenized fresh plant material using a solvent mixture of methanol, ethyl acetate, and petroleum ether in a 1:1:1 (*v*/*v*/*v*) ratio, following previously described protocol [81]. The extraction was repeated until the residue appeared colorless. The combined extracts were partitioned in a separation funnel with diethyl ether and a saturated saline solution. The organic phase was collected and concentrated to dryness under reduced pressure using a rotary evaporator. The resulting residue was dissolved in 10 mL of diethyl ether and saponified by mixing with an equal volume of 30% methanolic potassium hydroxide for 2 h. The mixture was transferred to a separation funnel and washed with saline solution until the aqueous phase reached a neutral pH. The organic phase was dried over anhydrous sodium sulfate and concentrated under a vacuum. The samples were stored at −20 °C until analysis.

Carotenoid quantification was performed by diluting the extracts with ethyl acetate, followed by filtration through a 0.45 µm PTFE membrane filter (VWR International, Radnor, PA, USA) and analysis using a Shimadzu HPLC system. The system was equipped with an LC-20AT binary pump (Prominence), a DGU-20A3 degasser (Prominence), and an SPD-M20 photodiode array detector (HPLC-PDA). Separation of carotenoids was performed on a YMC C30 column (24 cm × 4.6 mm, 5 µm particle size) using gradient elution with two solvent mixtures at a flow rate of 0.8 mL/min. Solvent A consisted of methanol/tert-butyl methyl ether/water (83:15:2, *v*/*v*/*v*), while Solvent B consisted of tert-butyl methyl ether/methanol/water (90:8:2, *v*/*v*/*v*). The gradient started with 1% Solvent B at 0 min and increased to 100% Solvent B by 160 min. Carotenoids were identified by comparing the UV-VIS spectra and retention times of sample peaks with those of standards, including β-carotene, lutein, and lycopene. The results were expressed as mg/100 g FW.

### 3.7. Statistical Analysis

The data were expressed as mean ± standard deviation (SD) from three replicates for each sample. The data analysis was carried out using a combination of software tools. Statistical analyses were performed with IBM SPSS Statistics (Version 26) and GraphPad Prism (Version 8). The Shapiro–Wilk test was used to assess the compatibility of the data with a normal distribution. One-Way ANOVA, an unpaired *t*-test, and Duncan’s multiple range tests (*p* < 0.0001, extremely significant ****; *p* = 0.0001 to 0.001, extremely significant ***; *p* = 0.001 to 0.01, very significant **; *p* = 0.01 to 0.05, significant *; *p* ≥ 0.05, insignificant ns) were performed to examine the differences among groups. In addition, a correlation matrix was generated using the corrplot package in the R software (Version 4.4.1, 2024) [82] to visualize the pairwise Pearson correlation coefficients among variables.

## 4. Conclusions

The foliar application of silicon nanoparticles (SiNPs) mitigated the harmful effects of increased UV-B radiation on tomato plants. Compared to UV-B-stressed controls, SiNP-treated foliage exhibited broader stomatal apertures and elevated intercellular CO_2_ levels. It enhanced net CO_2_ uptake without modifying transpiration, suggesting increased gas exchange efficiency and potential stabilization of chloroplast function. SiNPs also influenced secondary metabolism: whereas brief UV-B exposure resulted in very slight alterations in biogenic volatile organic compound emissions, leaves and fruits exhibited increased carotenoid accumulation, with a moderate but nutritionally significant rise in lycopene levels. These results collectively indicate that foliar SiNPs are a viable agronomic strategy for maintaining tomato yield and fruit quality under increased UV-B exposure.

However, the current study had several drawbacks. Experiments were performed in controlled-environment chambers, which are devoid of the multifaceted biotic and abiotic challenges present in open fields; hence, the efficiency of SiNPs in actual agronomic circumstances requires validation. Furthermore, just one UV-B regime and a singular SiNP dosage were investigated, leaving the response curves to broader irradiance and concentration ranges unexamined. The physiological scope was confined to gas exchange, pigments, and BVOCs; essential molecular, oxidative stress, and overall fruit quality indicators were not evaluated. The study did not encompass the long-term environmental and food safety effects of recurrent SiNP treatments. Future research should address these deficiencies by conducting multi-site field trials that incorporate transcriptomic, metabolomic, and toxicological assessments across varying UV-B intensities and SiNP dosages, thereby offering a comprehensive evaluation of the agronomic potential and safety profile of SiNPs in crop production.

## Data Availability

The raw data supporting the conclusions of this article will be made available by the authors on request.

## Supplementary Materials

The following supporting information can be downloaded at: https://www.mdpi.com/article/10.3390/plants14162599/s1, Figure S1. Measurement of photosynthetic parameters of tomato leaves using the GFS 3000 Portable Gas Exchange System; Figure S2. Tomato fruit harvested for lycopene and carotenoid content analysis.
