# Supplementary material for: Enhancing UV-B Protection and Abiotic Stress Tolerance in Tomato Plants: The Role of Silicon Nanoparticles in Photosynthetic Parameters, Pigments, and Secondary Metabolite Production
Source: Plants (Basel). 2025 Aug 21;14(16):2599. doi: 10.3390/plants14162599 (PMC12389229; doi:10.3390/plants14162599)
Supplement: Supplementary file 1 [file plants-14-02599-s001.zip › plants-3784412-supplementary.pdf]

plants

MDPI

Supplementary materials

Enhancing UV-B Protection and Abiotic Stress Tolerance in Tomato Plants: The Role of Silicon Nanoparticles in Photosynthetic Parameters, Pigments, and Secondary Metabolite Production

Florina Copaciu [1], Cosmin-Alin Faur [1], Andrea Bunea [1], Loredana Leopold [2], Rodica Maria Sima [3], Mihai Andrei Lăcătuș [1], Andreea Lupitu [4], Cristian Moisa [4], Dana Maria Copolovici [4] and Lucian Copolovici [4,*]

1 Faculty of Animal Science and Biotechnologies, University of Agricultural Sciences and Veterinary Medicine of Cluj-Napoca, 3-5 Manastur St., 400372 Cluj-Napoca, Romania
2 Faculty of Food Science and Technology, University of Agricultural Sciences and Veterinary Medicine, 3-5 Manastur St., 400372 Cluj-Napoca, Romania
3 Faculty of Horticulture and Business in Rural Development, University of Agricultural Sciences and Veterinary Medicine of Cluj-Napoca, 3-5 Manastur St., 400372 Cluj-Napoca, Romania
4 Institute for Interdisciplinary Research, Faculty of Food Engineering, Tourism and Environmental Protection, "Aurel Vlaicu" University of Arad, 2 Elena Dragoi St., 310330 Arad, Romania
* Correspondence: lucian.copolovici@uav.ro

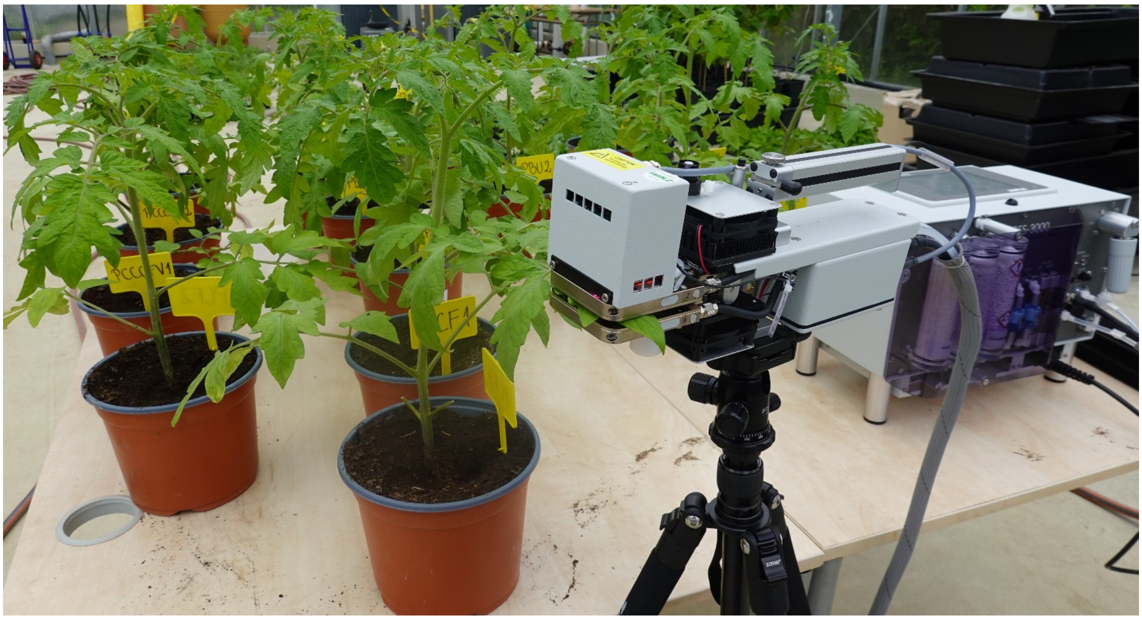

Figure S1. Measurement of photosynthetic parameters of tomato leaves using the GFS 3000 Portable Gas Exchange System.

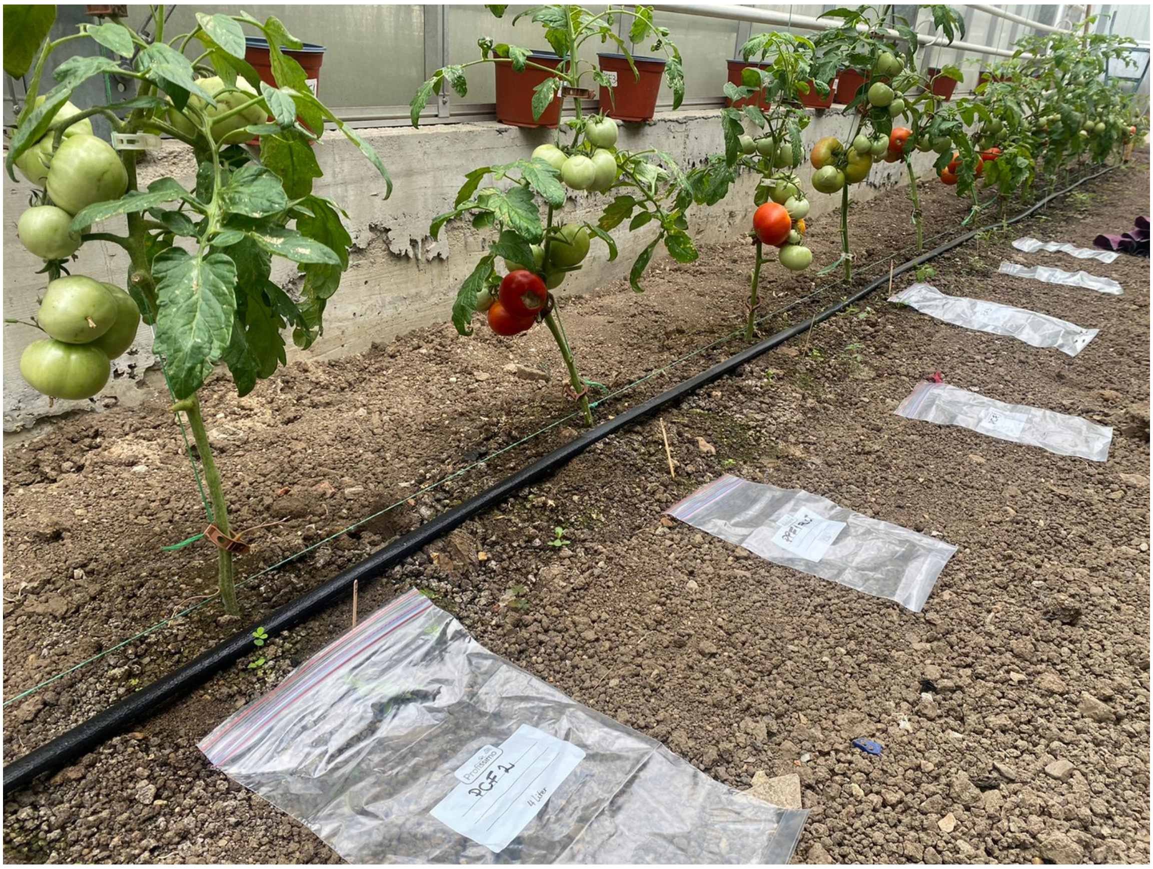

Figure S2. Tomato fruit harvested for lycopene and carotenoid content analysis.
